# A comprehensive analysis and validation of cuproptosis-associated genes across cancers: Overall survival, the tumor microenvironment, stemness scores, and drug sensitivity

**DOI:** 10.3389/fgene.2022.939956

**Published:** 2022-08-29

**Authors:** Jinsong Liu, Yueyao Lu, Yuyang Dai, Ying Shen, Cheng Zeng, Xiuling Liu, Huayi Yu, Jianzhong Deng, Wenbin Lu

**Affiliations:** ^1^ Department of Oncology, Wujin Hospital Affiliated with Jiangsu University, Jiangsu, China; ^2^ Department of Oncology, The Changzhou Clinical School of Nanjing Medical University, Jiangsu, China; ^3^ Department of Radiology, Wujin Hospital Affiliated with Jiangsu University, Changzhou, Jiangsu, China; ^4^ Department of Oncology, The Wujin Clinical College of Xuzhou Medical University, Jiangsu, China

**Keywords:** cuproptosis-associated genes, pan-cancer analysis, differential analysis, overall survival, immune subtype, the tumor microenvironment, stemness score, drug sensitivity

## Abstract

**Background:** Cuproptosis is a novel type of cell death induced by copper. Cuproptosis-associated genes play a crucial part in oncogenesis and the growth and metastasis of tumors. However, the correlations among cuproptosis-associated genes, overall survival, the tumor microenvironment, and drug sensitivity remain unclear. Therefore, we performed an analysis of cuproptosis-associated genes across cancers.

**Methods:** We downloaded RNA sequence expression data, clinical and survival data, stemness score data, and immune subtype data of cuproptosis-associated genes from the UCSC Xena. Next, we conducted differential analysis, expression analysis and correlation analysis across cancers with various R packages. Moreover, survival analysis and Cox hazard analysis were conducted to investigate the relationships between cuproptosis-associated genes and survival outcomes in various cancer types. Finally, we also analyzed the relationship among the levels of cuproptosis-associated genes across cancers, immune types, the tumor microenvironment, stemness scores, and drug sensitivity. Expression validation of cuproptosis-associated genes in renal cancer and normal tissues by immunohistochemical staining.

**Results:** We found that 10 cuproptosis-associated genes (FDX1, LIAS, LIPT1, DLD, DLAT, PDHA1, PDHB, MTF1, GLS, and CDKN2A) were differently expressed in 18 tumors and normal tissues. Survival outcomes showed that cuproptosis-associated genes had prognostic value in various cancer types. Moreover, we identified that cuproptosis-associated genes had different levels in six immune subtypes. The study also indicated that the levels of most cuproptosis-associated genes were positively correlated with the RNAss and DNAss. FDX1, LIAS, LIPT1, DLD, DLAT, PDHA1, and PDHB were negatively correlated with immune scores and ESTIMATE scores. In addition, we identified the top 16 drugs strongly sensitivity to cuproptosis-associated genes according to the correlation coefficient. Finally, we also found that cuproptosis-associated genes were significantly correlated with immune subtype, clinical features, the tumor microenvironment, and drug sensitivity in Kidney renal clear cell carcinoma. And the results of immunohistochemical staining analysis was very consistent with the previous analysis.

**Conclusion:** We performed an overall analysis to uncover the roles of cuproptosis-associated genes in differential expression, survival outcomes, immune subtypes, the tumor microenvironment, stemness scores, and cancer drug sensitivity across cancers.

## Introduction

Previously reported statistics have shown that cancer is the main public health problem globally and the second most common cause of death (Bray et al., 2021). In 2020, there were roughly 19.3 million new cases and 10 million deaths globally (Sung et al., 2021). Although there have been significant advances in diagnosis and therapy techniques, survival time of individuals with cancer remains extremely short. Therefore, it is significant and urgent to explore potential predictive indicators and promising therapeutic strategies.

There are many kinds of cell death, such as necroptosis, pyroptosis, autophagy, and ferroptosis (Tang et al., 2020). Previous study has found that after blocking adenosine triphosphate (ATP) biosynthesis, a high concentration of zinc can induce nonapoptotic cell death (Dineley et al., 2003; Du et al., 2021). Besides, ferroptosis, a unique kind of cell death, is characterized by the production of hazardous membrane lipid peroxides, which is catalyzed by iron (Kagan et al., 2017). Another trace metals, including copper, are necessary for a number of biological activities in living organisms. However, these trace metals should be present in cells in appropriate amounts. Insufficient metal concentrations can cause essential metal-binding enzymes to malfunction, while excessive metal concentrations can increase the cellular burden and cause cell death. In 1928, scientists investigated the role of copper in physiological processes for the first time (Tardito and Marchio, 2009). Cu-TSCs (thiosemicarbazones) were shown to have antitumor activity in the 1960s, pioneering a new era of cancer treatment with copper compounds (Booth and Sartorelli, 1966; Cappuccino et al., 1967). In 2005, scientists found that the production of reactive oxygen and nitrogen species was a fundamental element influencing the toxic effects and carcinogenicity of trace metals, including copper (Valko et al., 2005). In addition, the Fenton reaction, which generates superoxide radicals and hydroxyl radicals, is involved in the influence of copper on living organisms (Singh et al., 2021). Previous studies have shown that unbalanced intracellular copper levels influence tumorigenesis and the growth and metastasis of tumors, causing irreversible damage. In humans, excessive copper accumulation has two sides. On the one hand, it is life-threatening. On the other hand, intracellular copper accumulation can be used to selectively kill cancer cells (Ge et al., 2022).

A major discovery was recently published in *Science*, as an increased concentration of intracellular copper was found to induce the polymerization of mitochondrial lipoylated proteins and the instability of Fe-S cluster proteins, inducing a novel kind of cell death known as cuproptosis (Tsvetkov et al., 2022). Unlike all other known mechanisms that regulate cell death, cuproptosis, which is a recently discovered copper-induced cell death process, has attracted substantial attention and has become a research hotspot in the field of tumor therapy. First, *Tsvetkov* and colleagues identified whether intracellular copper triggered a new type of regulated cell death that was distinct from oxidative stress-associated cell death processes, such as ferroptosis, apoptosis, and necroptosis. They used different cell death inhibitors, including Z-VAD-FMK, ferrostatin-1, liproxstatin-1, and necrostatin-1, to suppress cell death triggered by copper complexes, but the inhibitors had no effect on the process. Second, it was demonstrated that copper-induced cell death required the involvement of mitochondrial respiration and that ATP produced by glycolysis had a minor effect on copper-induced cell death. Immediately afterward, the authors also found that copper was not directly involved in the electron transport chain (ETC) but played a role in only the tricarboxylic acid (TCA) cycle. Finally, the researchers found that the copper that penetrated into the mitochondria *via* copper carriers bound directly to these lipid-modified proteins, causing them to form long chains and clump together, leading to cell death. These copper molecules also interfered with iron-sulfur clusters, leading to the downregulation of iron-sulfur proteins and resulting in cytotoxic stress and death. This surprising discovery illustrated how copper actually disrupted mitochondrial function and how it caused cell death, which laid a solid foundation for elucidating the pathology of inherited copper overload illnesses and developing novel ways to treat cancer *via* copper toxicity.

In our work, we first downloaded RNA sequence expression data, clinical and survival data, stemness score data, and immune subtype data of cuproptosis-associated genes (FDX1, LIAS, LIPT1, DLD, DLAT, PDHA1, PDHB, MTF1, GLS, and CDKN2A) from the UCSC Xena. Next, we conducted differential analysis, expression analysis and correlation analysis across cancers. Moreover, survival analysis was conducted to identify the connection between the prognostic outcomes of cuproptosis-associated genes and various cancer types from TCGA. Additionally, we examined the relationship among the levels of cuproptosis-associated genes and immune types, the tumor microenvironment and tumor stem cells across cancers. We further explored the association between drug sensitivity and cuproptosis-associated genes. Finally, the relationship between cuproptosis-associated genes in Kidney renal clear cell carcinoma and tumor features was further analyzed in our work. And we also validated the protein expression by immunohistochemical staining. The above thorough research paved a path for discovering new functions of cuproptosis-associated genes and potential chemotherapy methods in pan-cancer research.

## Materials and methods

### Data downloading

RNA sequence expression data, clinical and survival data, stemness score data (based on DNA methylation and RNA expression), and immune subtype data of 33 TCGA tumors were abstracted from UCSC Xena (http://xena.ucsc.edu/) (Goldman et al., 2020; Miao et al., 2020). The abbreviations and full names of 33 tumors are listed in the Supplementary Table S1. Because the data from TCGA are open access, this study does not need the approval of the clinical ethics committee. The study complied with the access policies and publication rules of TCGA.

### Differential expression analysis and correlation analysis of various cancer types

We obtained 10 cuproptosis-associated genes (FDX1, LIAS, LIPT1, DLD, DLAT, PDHA1, PDHB, MTF1, GLS, and CDKN2A) from the article: copper induces cell death by targeting lipoylated TCA cycle proteins, which was recently published in *Science* (Tsvetkov et al., 2022). These 10 genes were abstracted from Figure 3A in the above article. The team used a genome-wide CRISPR-Cas9 to ensure the scientific and accurate screening and the final screen yielded only 10 genes. These 10 genes were involved in the direct regulation of the vital activity of copper death. Therefore, we selected 10 cuproptosis-associated genes for the following analysis. To study the overall expression of 10 cuproptosis-associated genes across cancers, we first generated a boxplot graph of the expression levels of cuproptosis-associated genes in 33 cancers. Moreover, we visualized the differential expression levels of 10 cuproptosis-associated genes in 18 tumor types, which contained over five normal samples. A heatmap was constructed utilizing the R package “pheatmap”. The Wilcoxon test was also used to explore the differences between normal and tumor tissues. Besides, the correlations of cuproptosis-associated genes with other parameters in 33 cancers were analyzed utilizing the R package “corrplot”. Finally, we performed Spearman’s test to analyze the differential levels of cuproptosis-associated genes in 18 tumors, which contained over five normal samples. Differential expression analysis was visualized with the R package “ggpubr”. *p < 0.05, **p < 0.01, and ***p < 0.001. *p* < 0.05 was considered statistically significant.

### Clinical correlation

To analyze the survival value of patients who had different levels of cuproptosis-associated genes, we performed survival analysis with the R packages “survival” and “survminer”. Additionally, Cox proportional hazard regression was also conducted to identify the connection between cuproptosis-associated genes and survival outcomes in various cancer types.

### Correlation analysis of six immune subtypes

We visualized the immune subtype analysis of cuproptosis-associated genes using the R packages “limma”, “ggplot2”, and “reshape2”. The Kruskal test was conducted to analyze the differential expression of cuproptosis-associated genes in six immune subtypes (C1: wound healing, C2: IFN-γ dominant, C3: inflammatory, C4: lymphocyte depleted, C5: immunologically quiet, and C6: TGF-β dominant) (Thorsson et al., 2018).

### Correlation analysis of the tumor microenvironment and tumor stem cells

To predict tumor purity and the infiltration of stromal and immune cells in various cancer types, we used the R packages “limma”, “estimate”, and “corrplot” to visualize the correlations between cuproptosis-associated genes and the tumor microenvironment (Diboun et al., 2006). Spearman’s test was used during the process. To analyze the features of tumor stem cells, we downloaded RNA expression data and DNA methylation data for various cancers from TCGA. RNA stemness score (RNAss) and DNA stemness score (DNAss) of cuproptosis-associated genes were presented using the R packages “limma” and “corrplot”. Spearman’s test was also used during the process.

### Drug sensitivity

The CellMiner database (https://discover.nci.nih.gov/cellminer/home.do) is a large database for collecting, processing, and integrating molecular data on NCI-60 and other tumor cells (Shankavaram et al., 2009; Reinhold et al., 2012). We first abstracted gene data and drug sensitivity data from the CellMiner. Drug sensitivity data were validated after a clinical trial and certified with FDA standards. Next, we conducted Pearson test to identify the connections between cuproptosis-associated genes and drug sensitivity. The process was visualized by using the R packages “impute”, “limma”, “ggplot2”, and “ggpubr”.

### Immune subtypes, clinical characteristics, stem cell scores and the tumor microenvironment in kidney renal clear cell carcinoma

To further identify the relationship between cuproptosis-associated genes and a specific cancer type (KIRC), we explored the levels of cuproptosis-associated genes in six immune subtypes using the Kruskal test. Moreover, we also identified the relationships between cuproptosis-associated genes and clinical characteristics, including stage, grade, age, and sex. Differential expression analysis of cuproptosis-associated genes and clinical characteristics was conducted with the R packages “limma”, “ggplot2”, and “reshape2”. The Kruskal test was also used in the process. Additionally, the correlations between stem cell stores and cuproptosis-associated genes were visualized by utilizing the R packages “limma”, “ggplot2”, “ggpubr”, and “reshape2”. Finally, we identified the connection between cuproptosis-associated genes and the tumor microenvironment with Spearman’s test.

### Validation analysis of the human protein atlas

The Human Protein Atlas (http://www.proteinatlas.org/) could be performed to map all the human proteins in tissues using an integration of antibody-based imaging. The tissue section and the pathology section show the distribution of the proteins across major normal tissues and cancer tissues, respectively. We verified the differences in gene expression at the protein level by comparing these two parts. There four degrees of staining: high, medium, low, and not detected.

### Statistical analysis

Statistical analyses were processed using the R package v. 3.6.3. The Wilcoxon test was used to explore the differences between normal and tumor tissues. The Kruskal test was conducted to analyze the differential expression of cuproptosis-associated genes in six immune subtypes. The connection between cuproptosis-associated genes and the tumor microenvironment with Spearman’s test. Pearson test was used in drug sensitivity analysis. *p < 0.05, **p < 0.01, and ***p < 0.001. *p* < 0.05 was considered statistically significant.

## Results

### The expression of cuproptosis-associated genes across cancers

**FIGURE 1:**
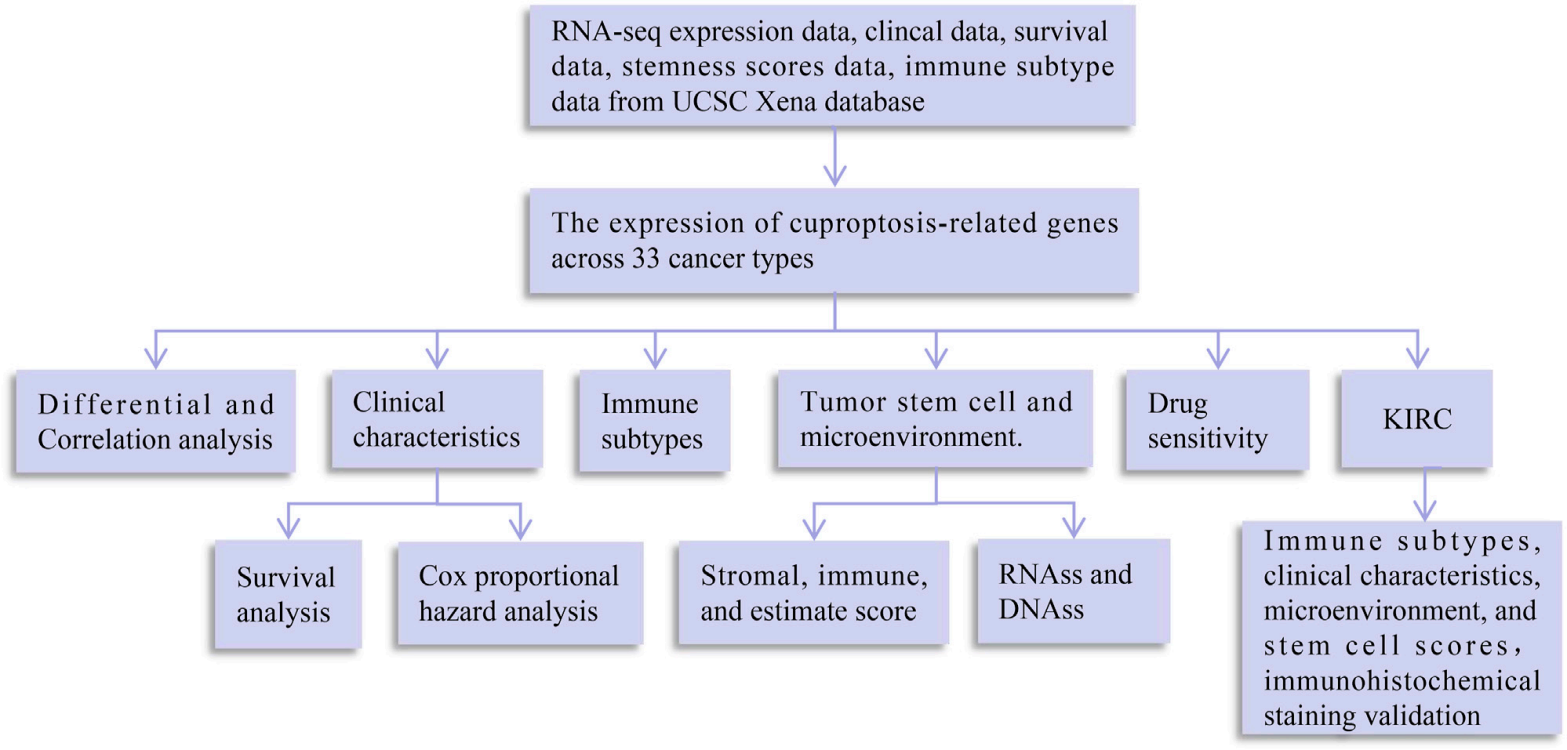
Flowchart of pan-cancer analysis in cuproptosis-associated genes. DNAss, DNA stemness score; RNAss, RNA stemness score.

**FIGURE 2:**
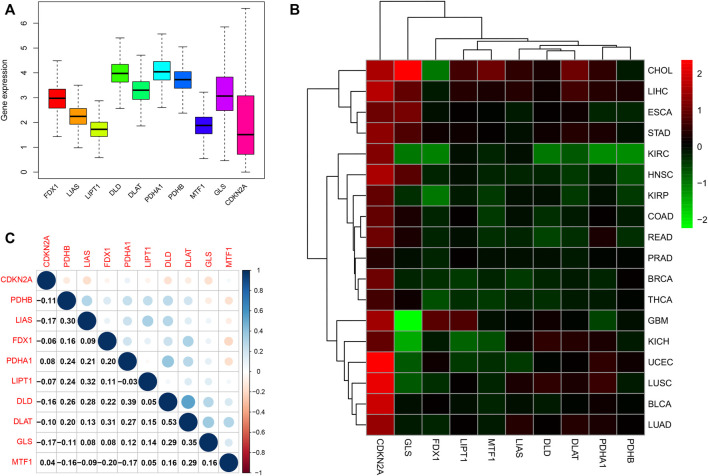
Differential expression and correlation analysis of 10 cuproptosis-associated genes in various cancer types. **(A)** Different levels of cuproptosis-associated genes in 33 cancers. Different colors in the bar chart represent different genes. The Y-axis in boxplot means the relative gene expression level in pan-cancer. **(B)** The differential expression of cuproptosis-associated genes in 18 cancers. The red boxes indicate high expression, and the green boxes indicate low expression. **(C)** Correlation analysis of cuproptosis-associated genes. Blue and red dots indicate positive and negative correlations, respectively.

**FIGURE 3:**
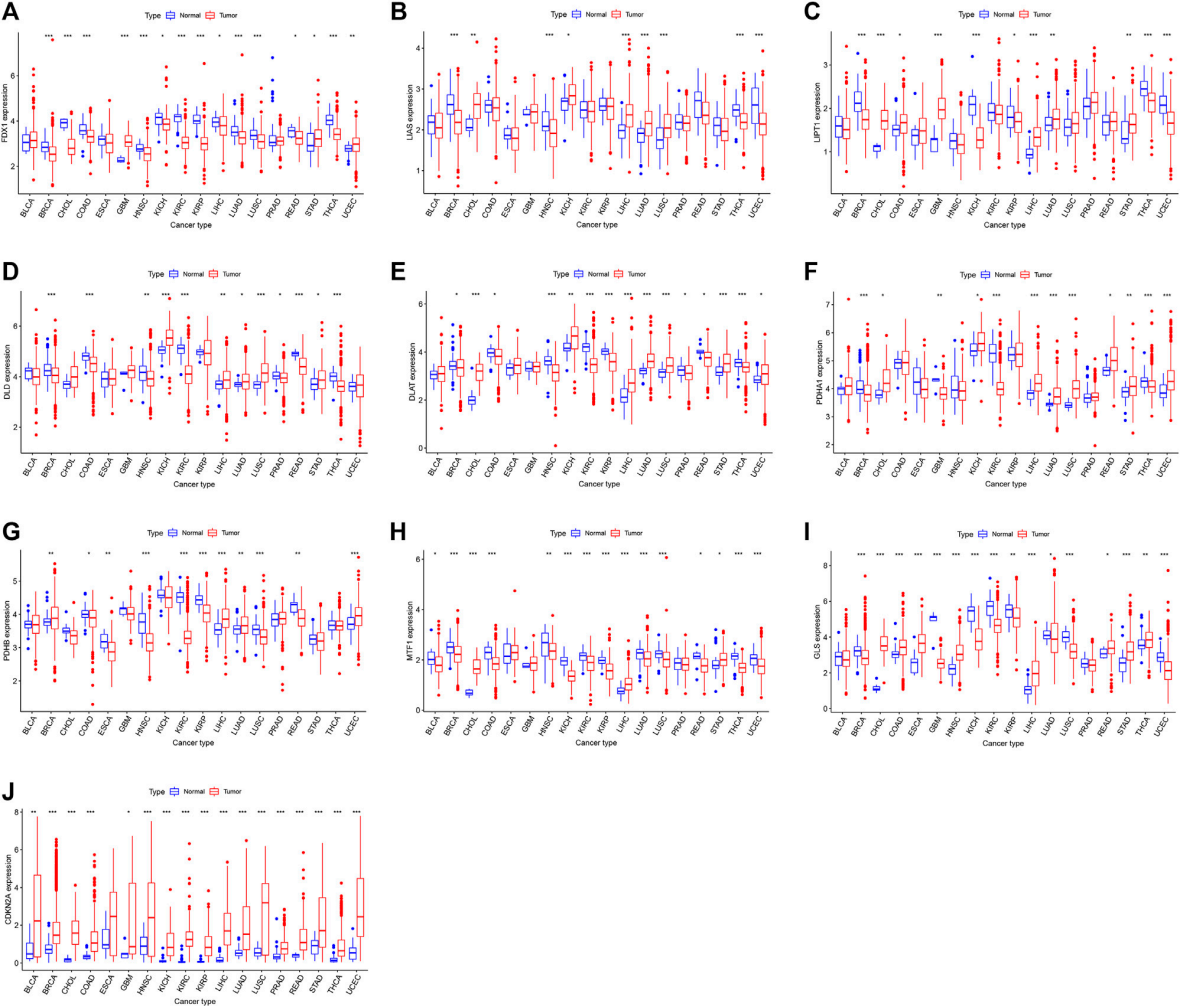
**(A–J)** The levels of cuproptosis-associated genes in various tumor and normal tissues. The blue box plot indicates normal tissue. The red box plot indicates tumor tissue. *p < 0.05, **p < 0.01, and ***p < 0.001.

A flowchart of pan-cancer analysis in cuproptosis-associated genes is shown in Figure 1. We first visualized the levels of 10 cuproptosis-associated genes in 33 cancers (Figure 2A). The boxplot shows that cuproptosis-associated genes, including FDX1, LIAS, DLD, DLAT, PDHA1, PDHB, GLS, LIPT1, MTF1, and CDKN2A had high expression in 33 cancers. Gene expression >1 means high expression level and Gene expression <1 means low expression level. Figure 2B shows the differential expression levels of cuproptosis-associated genes in 18 tumor types compared with adjacent non-tumor tissues. The heatmap shows that the fold change in cuproptosis-associated gene was greater than 1, which indicated that the level of cuproptosis-associated gene was higher in tumor tissue than corresponding normal tissue. It was surprise findings that CDKN2A had higher expression levels in 18 tumor types, which indicated that CDKN2A could be a novel biomarker in various cancers. Figure 2C presents the correlation analysis of cuproptosis-associated genes in 33 cancers. DLD and DLAT shared the strongest positive correlation (correlation coefficient = 0.53). However, FDX1 and MTF1 had the strongest negative correlation (correlation coefficient = −0.20). The expression of cuproptosis-associated genes in different tumors and normal tissues is presented in Figure 3. The boxplots indicates that CDKN2A had higher levels in various cancers, in accordance with the outcomes in Figure 2B.

### Prognostic value of cuproptosis-associated genes across cancers

**FIGURE 4:**
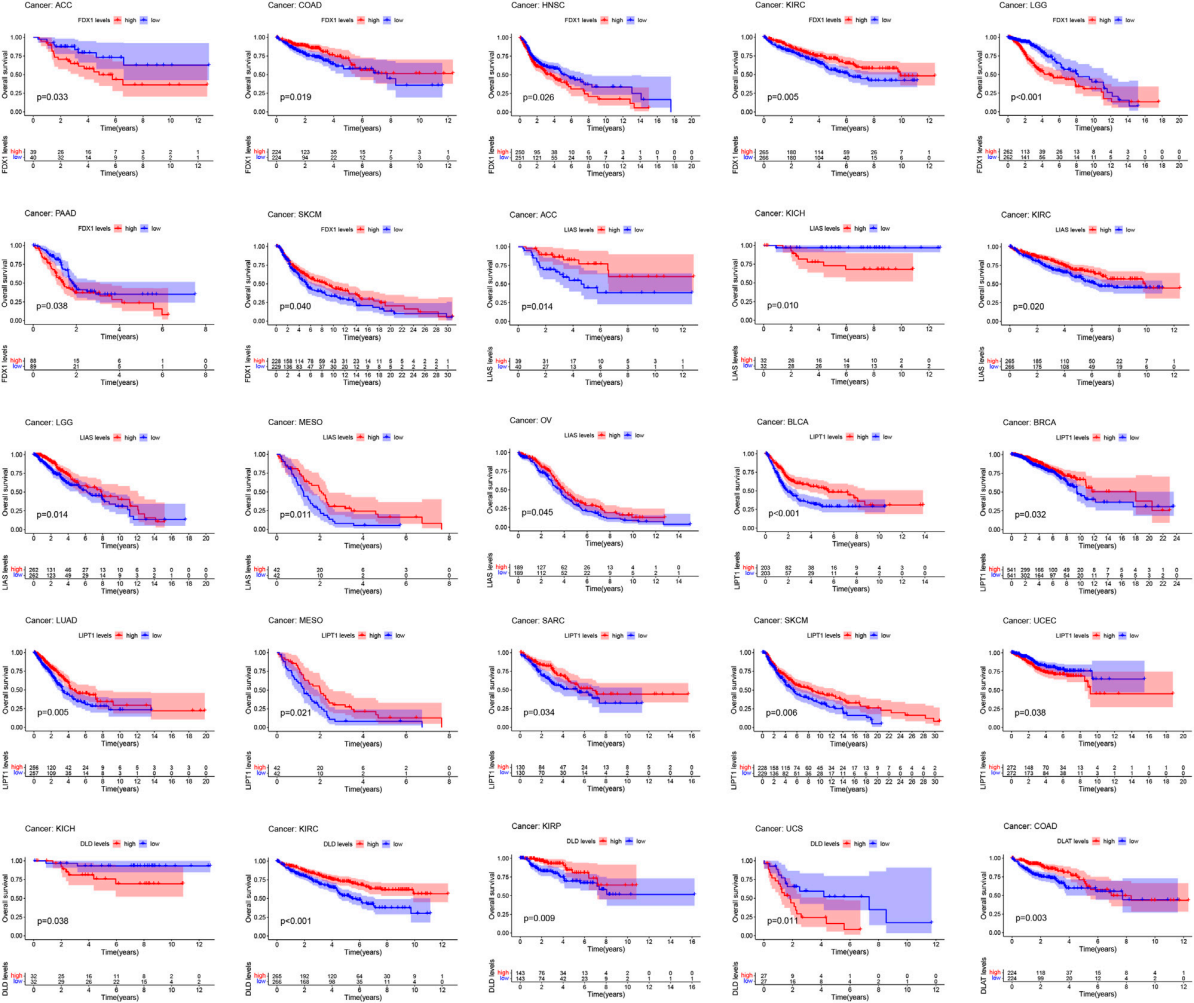
Survival analysis of cuproptosis-associated genes in various cancers. The red and blue lines of the picture indicate high and low level, respectively.

**FIGURE 5:**
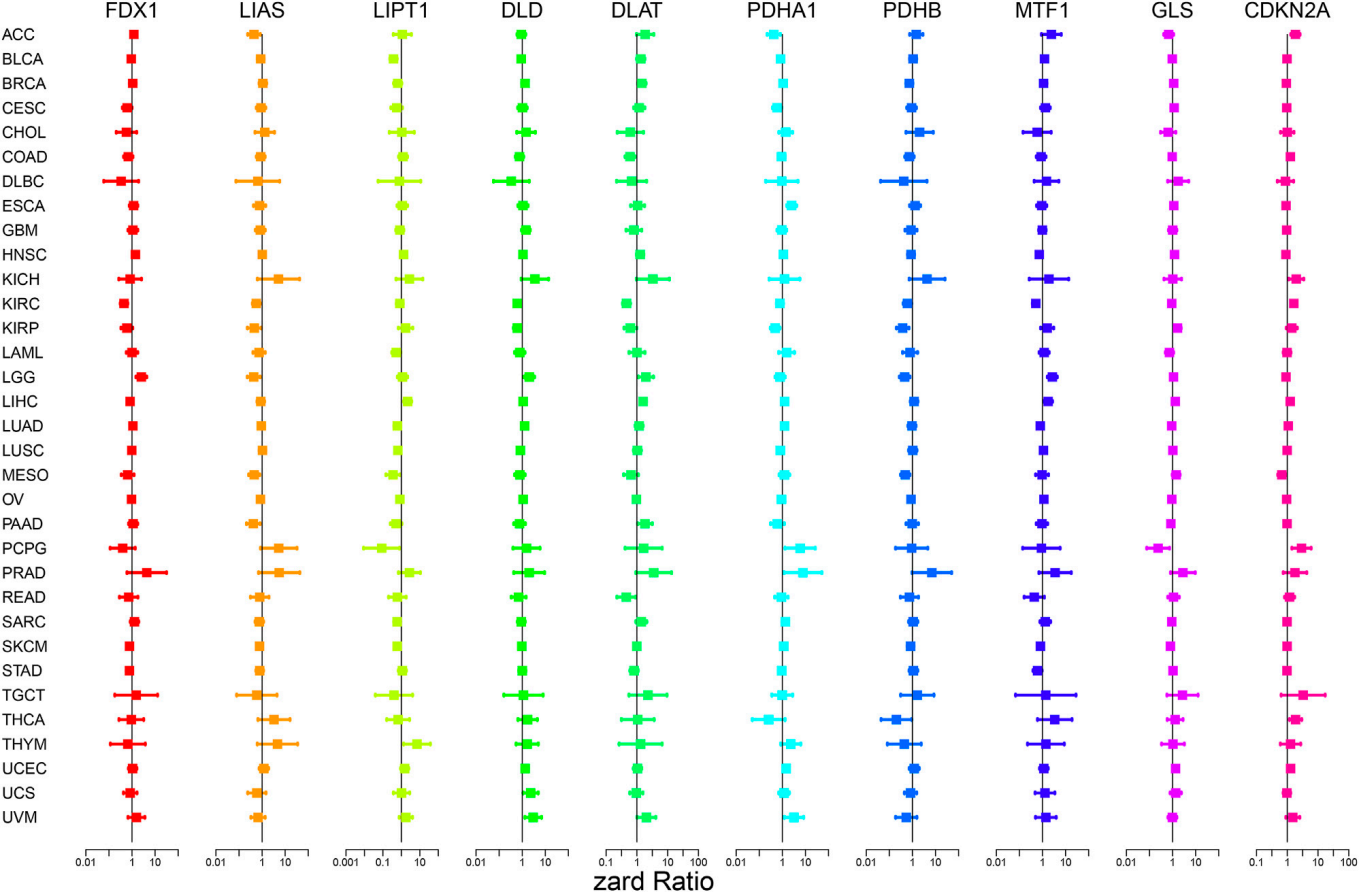
Cox proportional hazard analysis indicates the overall survival rate associated with cuproptosis-associated gene expression in various cancer types. A hazard ratio <1 indicates low risk, and a hazard ratio >1 indicates high risk.

Figure 4; Supplementary Figure S1 show the survival analysis of cuproptosis-associated genes in various cancers. A large majority of cuproptosis-associated genes including FDX1, LIAS, DLD, DLAT, MTF1, and CDKN2A had significant overall survival (*p* < 0.05), which indicated that cuproptosis-associated genes could be prognosis factors in Kidney renal clear cell carcinoma (KIRC). The above results also indicated that cuproptosis could play a crucial part in the occurrence and development of KIRC, paving the way for the following analysis. We also discovered that cuproptosis-associated genes including FDX1, LIAS, PDHA1, MTF1, CDKN2A had significant overall survival (*p* < 0.05), which indicated that cuproptosis-associated genes could be prognosis factors in Adrenocortical carcinoma (ACC). Similarly, cuproptosis-associated genes including LIAS, LIPT1, PDHB, GLS, and CDKN2A had significant overall survival in Mesothelioma (MESO) (*p* < 0.05). Cox proportional hazard analysis indicated the overall survival rates associated with cuproptosis-associated gene expression in various cancer types, as shown in Figure 5. And explicit HR and *p*-value in the Cox risk model were found in the Supplementary Table S2. A hazard ratio <1 indicated that cuproptosis-associated genes could be low risk factors; however, a hazard ratio >1 indicated that cuproptosis-associated genes could be high risk factors. As shown in the figure, all cuproptosis-associated genes were high risk factors in Prostate adenocarcinoma (PRAD). However, a large majority of cuproptosis-associated genes including FDX1, LIAS, LIPT1, DLD, DLAT, and PDHB acted as a low-risk indicator in Lymphoid neoplasm diffuse large B-cell lymphoma (DLBC). The above results indicated that cuproptosis-associated genes played a vital role in the survival of the patients with PRAD or DLBC.

### Correlation analysis of cuproptosis-associated genes and different immune subtypes

**FIGURE 6:**
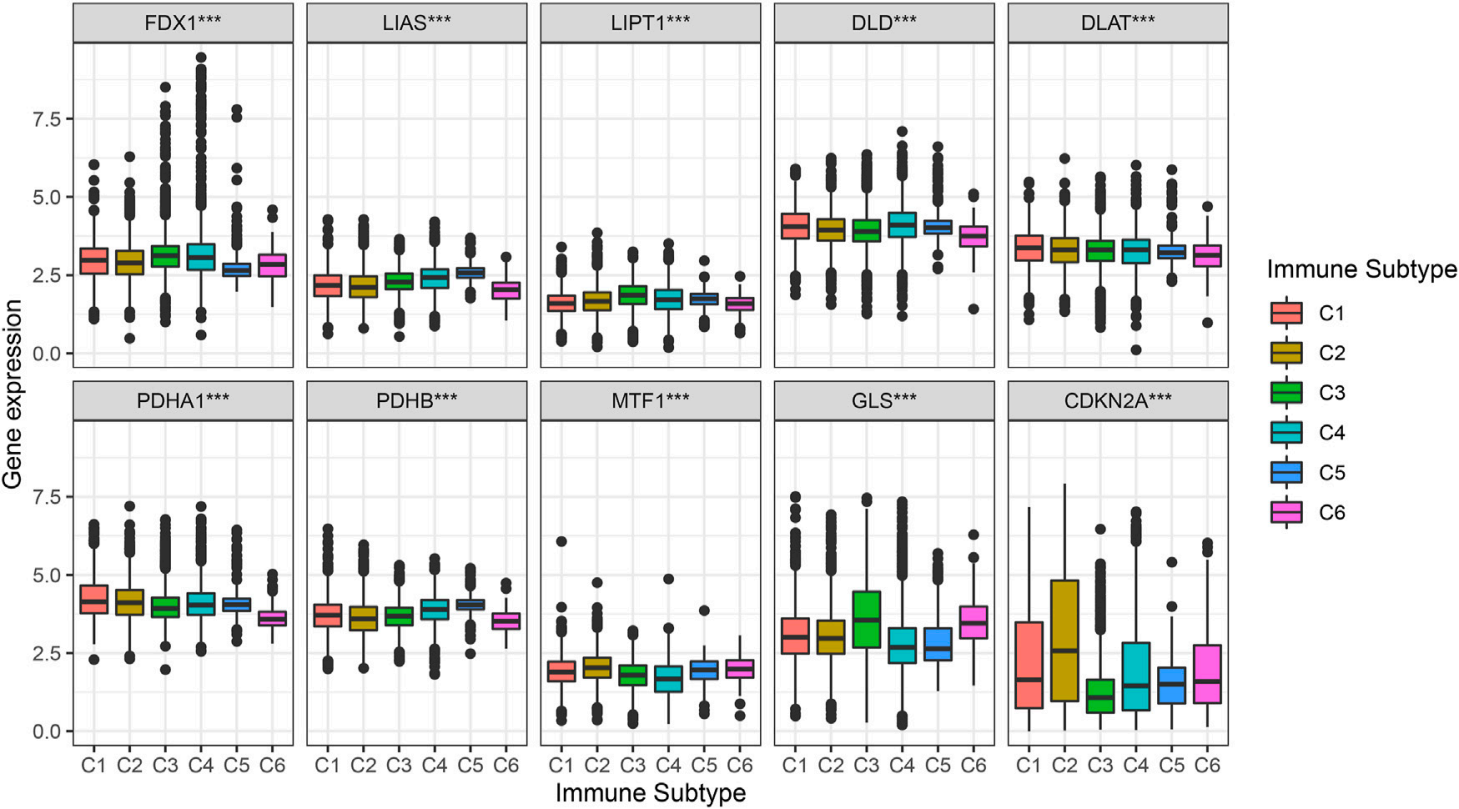
Correlation analysis between cuproptosis-associated genes and six immune subtypes. C1, wound healing, C2, IFN-γ dominant, C3, inflammatory, C4, lymphocyte depleted, C5, immunologically quiet, and C6, TGF-β dominant.

Figure 6 shows the different levels of cuproptosis-associated genes in six different immune subtypes. Cuproptosis-associated genes, including FDX1, LIAS, LIPT1, DLD, DLAT, PDHA1, PDHB, MTF1, and GLS were highly expressed in different immune subtypes. The levels of CDKN2A were higher in C1, C2, C4, C5, and C6, and lower in C3. *p*-value is labeled as the comparison of six different immune subtypes.

### Correlation analysis among cuproptosis-associated genes, stem cell scores and the tumor microenvironment

**FIGURE 7:**
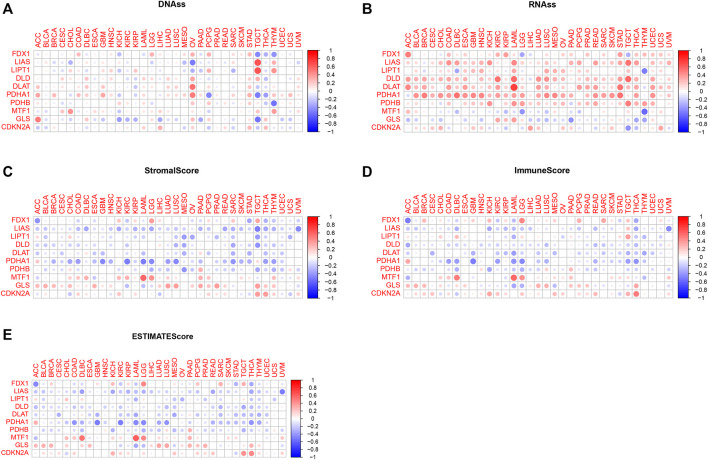
Correlation analysis of cuproptosis-associated genes with tumor stem cells and the tumor microenvironment. **(A)** Correlation analysis between cuproptosis-associated gene expression and stemness scores based on DNA methylation. **(B)** Correlation analysis between cuproptosis-associated gene expression and stemness scores based on RNA expression. **(C–E)** The relationships among the expression of cuproptosis-associated genes and stromal scores, immune scores, and ESTIMATE scores.

Tumor cells can lose their differentiated phenotype and develop precursor and stem cell-like characteristics as they develop. RNAss and DNAss were calculated to assess the features of tumor stem cells (Malta et al., 2018). Correlation analysis of cuproptosis-associated gene and stemness scores is shown in Figures 7A,B. As shown in the picture, the expression of a large majority of cuproptosis-associated genes including FDX1, LIAS, LIPT1, DLD, DLAT, PDHA1, and PDHB was positively correlated with RNAss and DNAss, which indicated that the higher the levels of cuproptosis-associated genes are, the higher the levels of stem cell scores, the more vigorous the tumor stem cells, and the lower the levels of tumor differentiation. However, MTF1 and GLS were negatively correlated with RNAss and DNAss. Figures 7C–E shows the associations among the expression of cuproptosis-associated genes and stromal, immune, and ESTIMATE scores. As shown in the picture, a large majority of cuproptosis-associated genes, including FDX1, LIAS, LIPT1, DLD, DLAT, PDHA1, and PDHB were negatively associated with immune, stromal, and estimated scores, which implies that the levels of immune and stromal cells are low across cancers and that tumor purity is high. However, some cuproptosis-associated genes, including MTF1, GLS, and CDKN2A, were positively associated with immune, stromal, and ESTIMATE scores, and the levels of immune and stromal cells were high across cancers, with low tumor purity.

### The relationship between cuproptosis-associated genes and drug sensitivity

**FIGURE 8:**
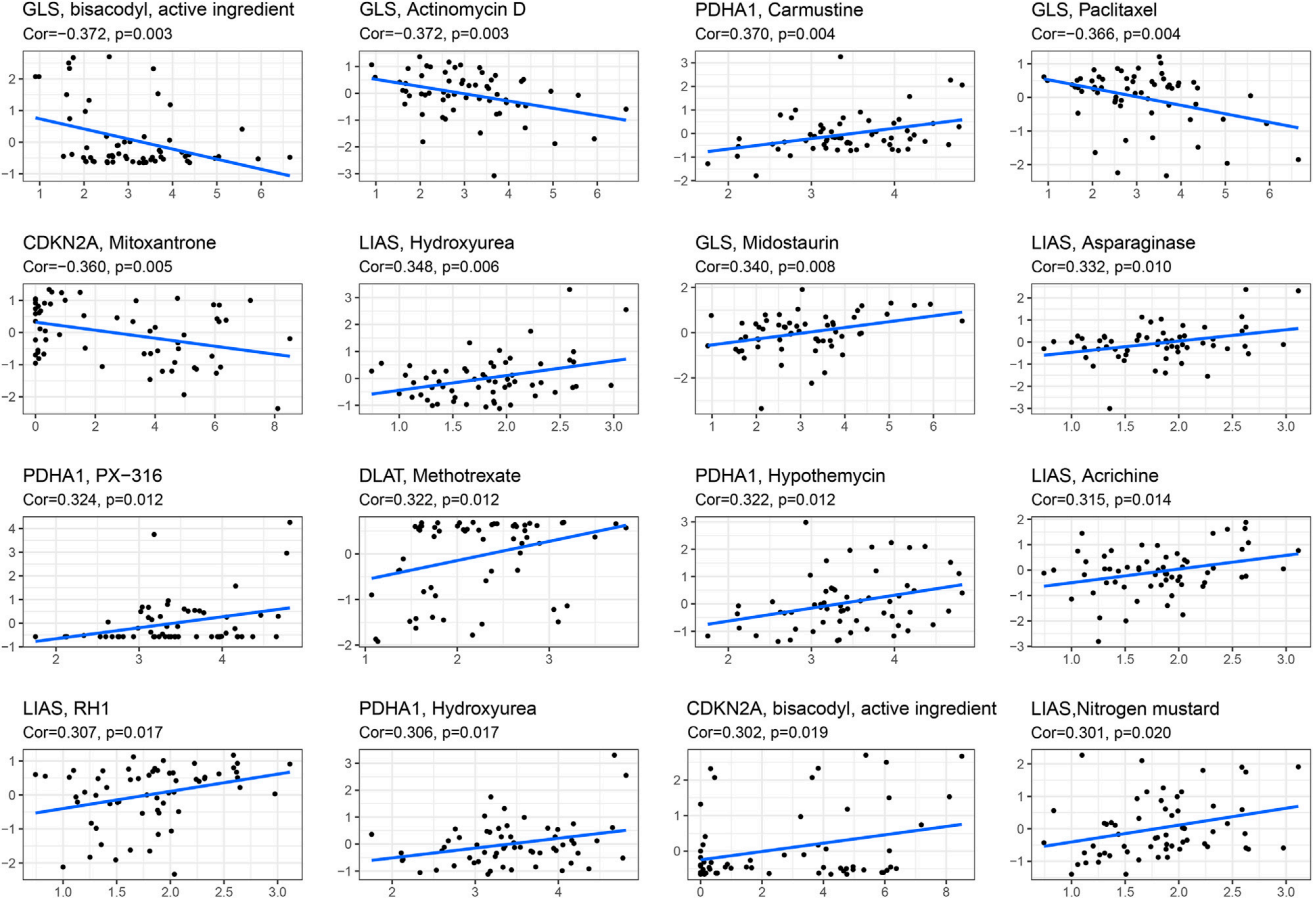
The relationship between cuproptosis-associated genes and drug sensitivity.

To explore the correlation between drug sensitivity and cuproptosis-associated genes, we conducted Pearson correlation analysis to address the expression data of cuproptosis-associated genes and drug sensitivity data. Figure 8 shows the top 16 drugs strongly sensitivity to cuproptosis-associated genes according to the correlation coefficient. As shown in the picture, GLS was negatively associated with sensitivity to bisacodyl, active ingredient of viraplex (cor = −0.372, *p* = 0.003), Actinomycin D (cor = −0.372, *p* = 0.003), Paclitaxel (cor = −0.366, *p* = 0.004) and positively associated with sensitivity to Midostaurin (cor = 0.340, *p* = 0.008). The higher the level of LIAS is, the stronger the sensitivity to drugs, including Hydroxyurea (cor = 0.348, *p* = 0.006), Asparaginase (cor = 0.332, *p* = 0.010), Acrichine (cor = 0.315, *p* = 0.014), RH1 (cor = 0.307, *p* = 0.017), and Nitrogen mustard (cor = 0.301, *p* = 0.020). PDHA1 was positively associated with Carmustine (cor = 0.370, *p* = 0.004), PX-316 (cor = 0.324, *p* = 0.012), and Hypothemycin (cor = 0.322, *p* = 0.012). The increased expression of CDKN2A resulted in less sensitivity to Mitoxantrone (cor = −0.360, *p* = 0.005) and stronger sensitivity to bisacodyl, active ingredient of viraplex (cor = 0.302, *p* = 0.019). DLAT and Methotrexate had positive correlation (cor = 0.322, *p* = 0.012). Cor means correlation coefficient.

### Correlation analysis of cuproptosis-associated genes, immune subtypes, and clinical features in kidney renal clear cell carcinoma

**FIGURE 9:**
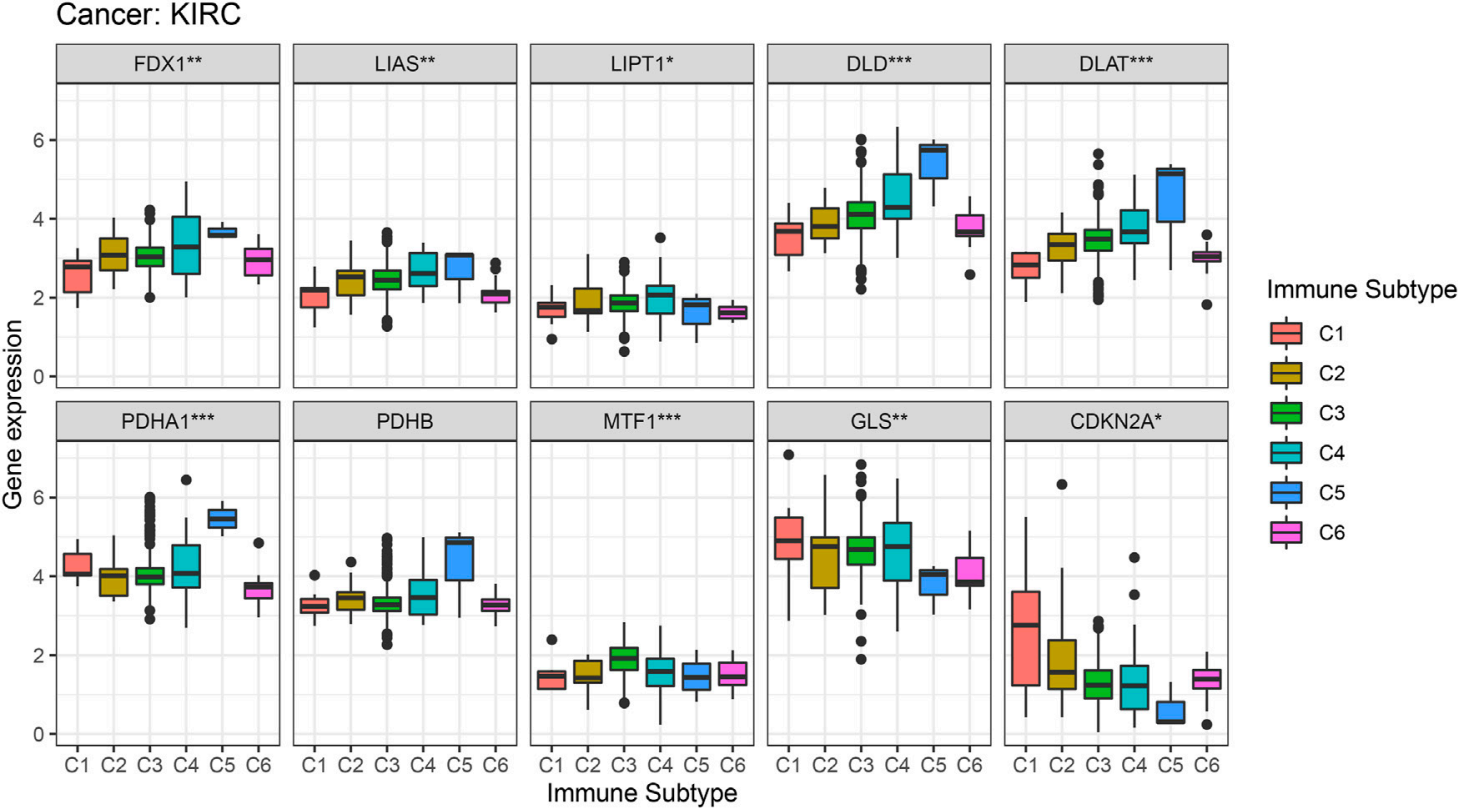
Correlation analysis between the expression of cuproptosis-associated genes and different immune subtypes in Kidney renal clear cell carcinoma (KIRC).

**FIGURE 10:**
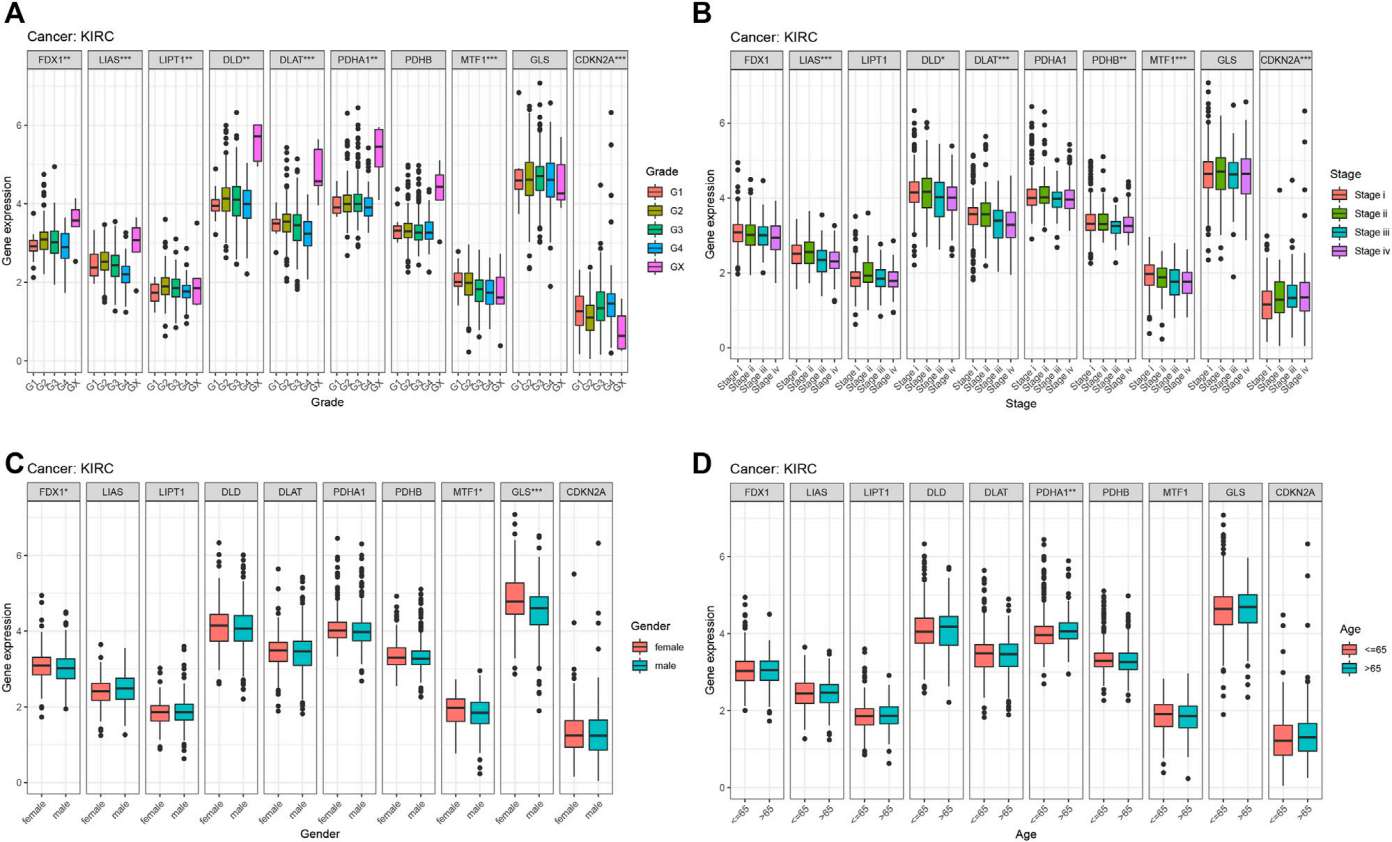
The relationship between cuproptosis-associated genes and clinical characteristics in KIRC. **(A)** Stage, **(B)** grade, **(C)** age, and **(D)** sex.

From previous survival analysis, we found that the number of cuproptosis-associated genes with survival statistical significance was the highest in KIRC, compared with other cancers, which indicated that cuproptosis could play a crucial part in the occurrence and development of KIRC. Therefore, we further analyzed the connection of cuproptosis-associated genes, immune subtypes and clinical features. Figure 9 shows the correlation of cuproptosis-associated genes and different immune subtypes in KIRC. All cuproptosis-associated genes except for PDHB, were associated with six immune subtypes, with statistical significance, and most of them had high expression in various immune subtypes. The correlation between cuproptosis-associated gene expression and clinical characteristics in KIRC is shown (Figure 10). Clinical features, including age, sex, stage, and grade, were studied in our work. Figure 10A shows that the expression levels of all cuproptosis-associated genes except for GLS were significantly different in different grades. Most of cuproptosis-associated genes including LIAS, DLD, DLAT, PDHA1, PDHB, MTF1, and CDKN2A had significantly differential expression according to stage (Figure 10B). The above results indicated cuproptosis had strong guidance value in clinical features of the patients with KIRC. The expression levels of FDX1, MTF1, and GLS varied significantly in different genders **(** Figure 10C). Figure 10D shows that only PDHA1 was associated with ages. *p*-value was the result of an inter-group comparison.

### The relationship of cuproptosis-associated genes, stem cell scores and the tumor microenvironment in kidney renal clear cell carcinoma

**FIGURE 11:**
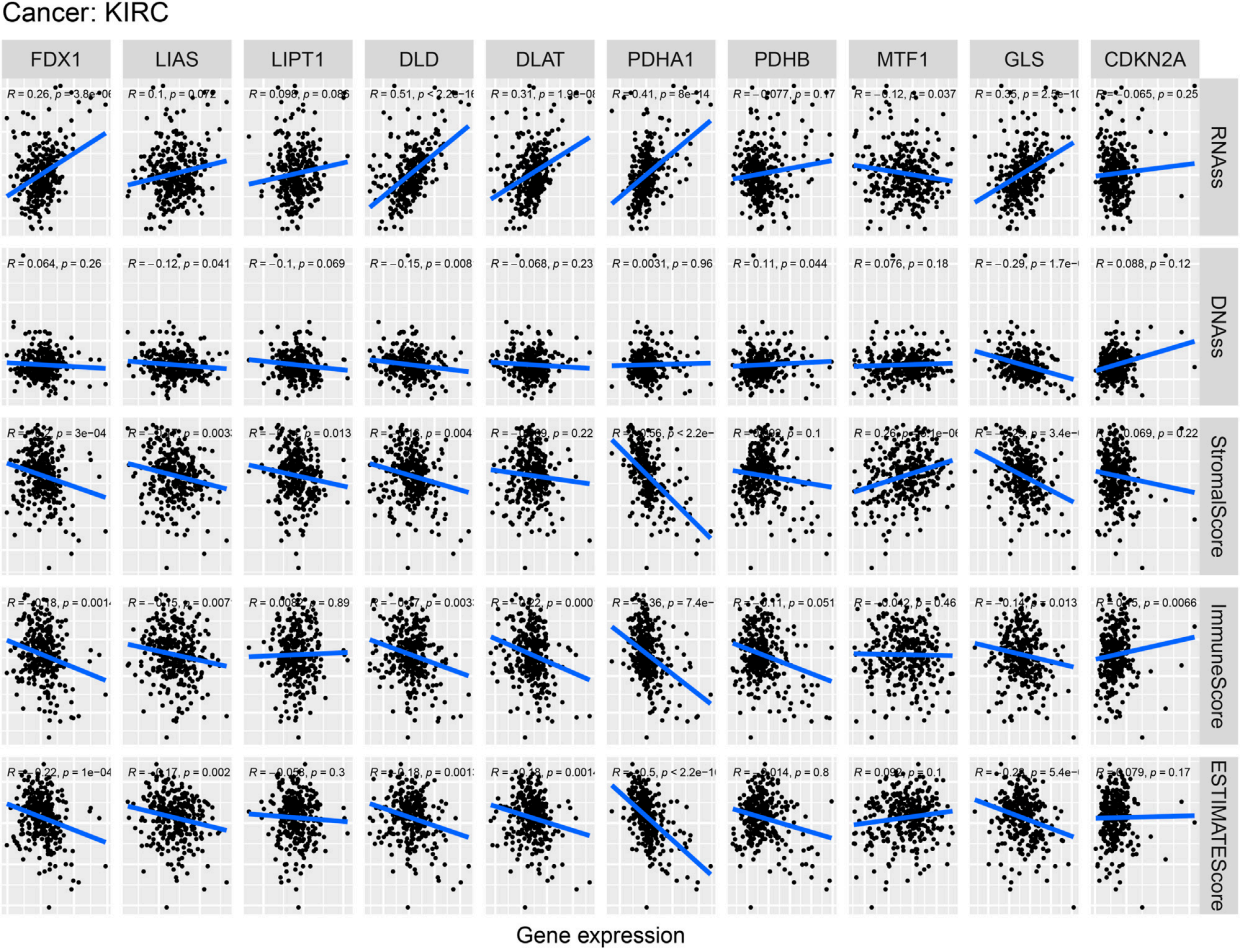
Correlation analysis of cuproptosis-associated gene expression, the tumor microenvironment, and stem cell scores in KIRC.

Figure 11 indicates the correlations of cuproptosis-associated genes, the KIRC microenvironment, and stem cell scores. Cuproptosis-associated genes, including DLD and GLS, were significantly associated with stemness scores (DNAss and RNAss) (*p* < 0.05). The scatter plot shows that the levels of DLD and GLS were positively associated with the DNAss and RNAss, which indicated that the higher the levels of DLD and GLS were, the higher the levels of stem cell scores, the more vigorous the tumor stem cells, and the lower the levels of tumor differentiation. Meanwhile, a large majority of cuproptosis-associated genes including FDX1, LIAS, DLD, DLAT, PDHA1, and GLS, were significantly associated with the tumor microenvironment (immune, stromal, and estimate scores) (*p* < 0.05). We surprisingly found that the levels of most of cuproptosis-associated genes were negatively associated with immune, stromal, and ESTIMATE scores, which implied that the lower the expression of cuproptosis-associated genes was, the higher the tumor purity. The above results implied that cuproptosis acted as a crucial role in the occurrence and development, which indicated that cuproptosis-associated genes could serve as a potential novel target for KIRC therapy.

### Validation of cuproptosis-associated genes in renal cancer tissues compared with normal tissues by immunohistochemical staining

**FIGURE 12:**
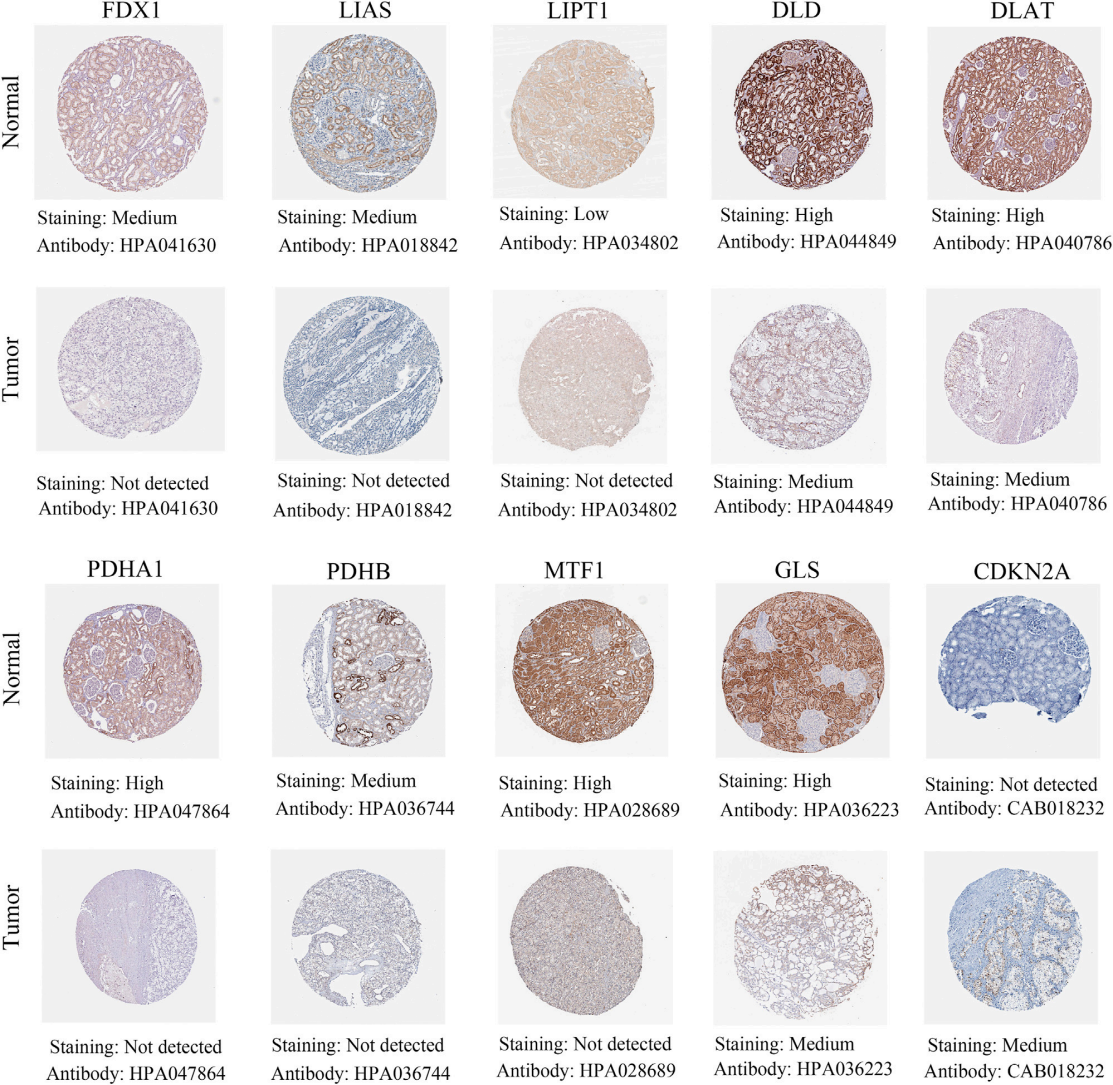
Different protein levels of cuproptosis-associated genes in renal cancer tissues compared with normal tissues by immunohistochemical staining.

The above results showed that differential expression levels of cuproptosis-associated genes played a crucial in survival outcomes, immune subtypes, clinical features, stem cell scores and the tumor microenvironment of patients. Next, by immunohistochemical staining, we identified protein expression levels of cuproptosis-associated genes in renal cancer tissues and normal tissues. The results demonstrated that a large majority of cuproptosis-associated genes including FDX1, LIAS, LIPT1, DLD, DLAT, PDHA1, PDHB, MTF1, and GLS had higher expression in normal tissues compared with renal cancer tissues, whereas CDKN2A was highly expressed in renal cancer tissues, which was very consistent with the results of our previous analysis (Figure 12).

## Discussion

Previous studies have shown that copper can induce various cell death pathways, such as apoptosis and autophagy, through different mechanisms, including an increase in reactive oxygen species, proteasome suppression, and antiangiogenesis (Jiang et al., 2022). The first mechanism is oxidative stress: Increased levels of ROS are triggered by the copper-regulated Fenton reaction or a deficiency of antioxidant suppression, which leads to mitochondrial malfunction and cell death. Second, copper bound to proteasome subunits, leading to the accumulation of ubiquitinated proteins. Third, copper deficiency restricted the creation of new blood vessels, reducing nutrient access in tumor tissues. A major discovery was recently published in *Science*: Copper induces cell death by targeting lipoylated TCA cycle proteins (Tsvetkov et al., 2022). This significant discovery fully illuminated how copper caused mitochondrial dysfunction and how this led to cell death (Zischka et al., 2011; Bock and Tait, 2020). These findings could imply that it is of great significance to maintain the dynamic balance of copper in the organism. Most importantly, these new outcomes could lay a solid foundation for investigating the usage of copper to treat cancer. Despite these significant advancements, scientists must increase awareness of the detailed mechanisms and effects of cuproptosis in the future (Kahlson and Dixon, 2022).

Cuproptosis-associated genes play a key part in the oncogenesis, progression and metastasis of tumors (Tang et al., 2022). Therefore, we performed an overall analysis to uncover the value of cuproptosis-associated genes across cancers. We obtained 10 cuproptosis-associated genes (FDX1, LIAS, LIPT1, DLD, DLAT, PDHA1, PDHB, MTF1, GLS, and CDKN2A) from the article: copper induces cell death by targeting lipoylated TCA cycle proteins, which was recently published in *Science* (Tsvetkov et al., 2022). Tsvetkov et al. firstly demonstrated that cuproptosis, a novel cell death path induced by copper ionophore, was distinguish from apoptotic, ferroptotic, necroptotic. Moreover, the research identified that copper-induced cell death was closely associated with mitochondrial metabolism, further elucidating the precise relationship between copper and the TCA cycle. Next, the team used a genome-wide CRISPR-Cas9 screen in order to clarify the specific metabolic pathway of this cell death and treated with different structures of copper ion vectors to improve the generality of the screen. The final screen yielded 10 genes, and the knockdown of each of these 10 genes significantly inhibited both types of copper ion vector-mediated cell killing. They further demonstrated that FDX1, the key gene for copper death, was also an upstream regulator of protein lipoic acidification. Besides, FDX1, LIAS, LIPT1, and DLD was involved in the lipoic acid pathway, and DLAT, PDHA1, PDHB, MTF1, GLS, and CDKN2A was involved in forming the Pyruvate dehydrogenase complex. The above genes were involved in the direct regulation of the vital activity of copper death. A genome-wide CRISPR-Cas9 screen is superior to other screening routes including correlation coefficients, Reason is that based only on correlation coefficients, the above genes are not representative and also not involved in the direct regulation of cuproptosis. Therefore, we selected 10 cuproptosis-associated genes from a genome-wide CRISPR-Cas9 screen for the following analysis.

Our work aimed to explore the relationships between cuproptosis-associated genes and tumor features, including clinical characteristics, survival outcome, immune subtype, tumor microenvironment, stemness score, and drug sensitivity. We found that all cuproptosis-associated genes, including FDX1, LIAS, LIPT1, DLD, DLAT, PDHA1, PDHB, MTF1, GLS, and CDKN2A had higher expression in various cancers. Our research revealed the differential expression levels of cuproptosis-associated genes in 18 cancer types compared with adjacent non-tumor tissues. Moreover, survival analysis showed that cuproptosis-associated genes had prognostic value in various cancer types. Cox proportional hazard analysis showed the overall survival rate of cuproptosis-associated genes in various cancer types, which indicated whether cuproptosis-associated genes were low- or high-risk factors. These outcomes strongly suggested that cuproptosis-associated genes could be significant diagnostic or prognostic markers across cancers, thereby contributing to research on targeted therapy.

The immune subtypes of cancers differ from one another, and each immune subtype has distinct biological and clinical characteristics that influence anticancer therapy to some extent. Our study also investigated the associations of cuproptosis-associated genes, immune subtype and clinical features. The findings showed that most of cuproptosis-associated genes, including FDX1, LIAS, LIPT1, DLD, DLAT, PDHA1, PDHB, MTF1, and GLS were highly expressed in different immune subtypes. The levels of CDKN2A were higher in C1, C2, C4, C5, and C6, and lower in C3. Previous research showed that tumors with the C4 and C6 subtypes had the worst prognosis, presenting with mainly macrophages, low lymphocyte infiltration, and a large number of M2 macrophages. In contrast, tumors consisting of the C2 and C3 subtypes had better survival outcomes. These findings implied that neoantigen could provide more prognostic signals in immune subtypes compared with tissue-based sources and highlight the significance of immune information for the response to tumor neoantigens.

The tumor microenvironment has a close relationship with oncogenesis and the growth and metastasis of tumors, and it is not only associated with the intrinsic environment of the tumor cells themselves (nuclear and cytoplasmic) but also includes various normal cells, including fibroblasts, vascular endothelial cells, stromal and immune cells (Neal et al., 2018; Jiao et al., 2019). We demonstrated that cuproptosis-associated genes were differentially associated with immune, stromal, and ESTIMATE scores, which revealed the effects of different tumor purities and infiltration of immune and stromal cells (Yoshihara et al., 2013). More importantly, the infiltration of immune and stromal cells was strongly associated with clinical outcomes and provided crucial clues for the diagnosis and prognosis of tumors. This infiltration was used to identify an effective drug target, further improving the survival outcome of patients (Malta et al., 2018). In addition, we also paid attention to the tumor stemness score. A higher stemness score was associated with stronger biological activity and weaker tumor dedifferentiation ability in tumor stem cells. Our work showed that a large majority of cuproptosis-associated genes was positively associated with RNAss and DNAss. These findings showed that the expression of cuproptosis-associated genes and the stemness score could predict the effectiveness of stem cell-associated immunotherapy, further improving the survival outcome of patients.

Finally, we investigated the interaction between drug sensitivity and cuproptosis-associated genes. The expression data of cuproptosis-associated genes and drug sensitivity data were abstracted from CellMiner, which included 108 FDA-approved and 70 clinical trial drugs and genomic data. Across numerous cancer types, this database provided a resource for pharmacologic data that could be used to strengthen existing therapeutic strategies and find new medications and pharmacological targets. Our findings revealed that cuproptosis-associated genes were closely associated with sensitivity to FDA-approved and clinical trial drugs. For example, both LIAS and PDHA1 were positively associated with sensitivity to Hydroxyurea. A recent study showed that some drugs, including Hydroxyurea, have shown promising anticancer efficacy *in vitro*, *in vivo*, and in clinical trials, making them potential candidates for therapeutic repurposing in oncology. These findings showed that cuproptosis-associated genes were associated with sensitivity to drugs and that their expression is important in the clinic for the selection of anticancer drugs. For certain cancers (KIRC), we also found that cuproptosis-associated genes were tightly associated with immune subtype, clinical features, the tumor microenvironment, and stem cell scores. And we also validated the protein expression by immunohistochemical staining.

We performed a pan-cancer analysis of cuproptosis-associated genes, which included differential expression, survival outcomes, immune subtypes, clinical features, the tumor microenvironment, stemness scores, and drug sensitivity. There was still some work to be done. First, we should further explore the mechanism of copper death in depth, and how cell respiration promotes cuproptosis. Second, we also should conduct a pan-cancer analysis of cuproptosis-associated genes with corresponding experiments for validation.

## Conclusion

In conclusion, this is the first and most comprehensive work to investigate the correlations between cuproptosis-associated genes and tumor features, including differential expression, survival outcomes, immune subtypes, the tumor immune microenvironment, stemness scores, and cancer drug sensitivity, which provides new insight into the mechanism of cuproptosis-associated genes across cancers and lays a solid foundation for the discovery of novel therapeutic targets.

## Data Availability

The original contributions presented in the study are included in the article/Supplementary Material, further inquiries can be directed to the corresponding author.

## Abbreviations

TCGA, The Cancer Genome Atlas; DNAss, DNA stemness score; RNAss, RNA stemness score.
