# Supplementary figures and images for: A comprehensive analysis and validation of cuproptosis-associated genes across cancers: Overall survival, the tumor microenvironment, stemness scores, and drug sensitivity
Source: Front Genet. 2022 Aug 29;13:939956. doi: 10.3389/fgene.2022.939956 (PMC9465292; doi:10.3389/fgene.2022.939956)

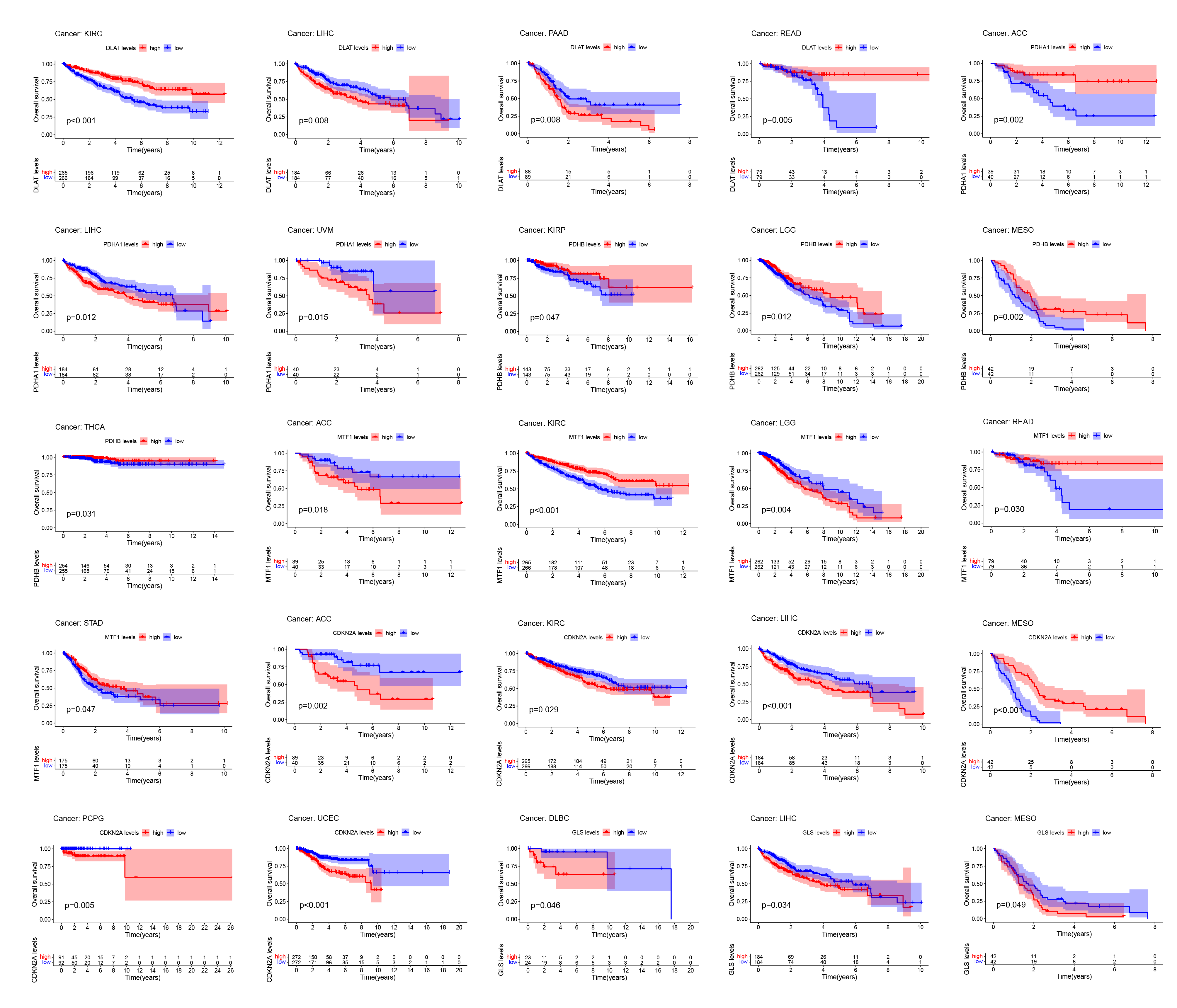

Supplement: Supplementary file 3 [file Image1.TIF]
